# Buffalo Academy of Medicine

**Published:** 1908-11

**Authors:** 


### SOCIETY PROCEEDINGS.

### Buffalo Academy of Medicine.

**Report of the Commission of Five, appointed for the purpose of investigating the conditions in Buffalo in regard to the care of contagious diseases and the need of a municipal hospital for contagious diseases.**

Your commission begs to report that from January 1, 1907, to December 31, 1907, there were reported to the Health Department of Buffalo communicable diseases as follows: measles, 2,388; scarlet fever, 1,324; diphtheria, 440; and the following number of deaths: measles, 46; scarlet fever, 59; diphtheria, 65; distributed through the different months as per attached table. There were per 10,000 inhabitants, measles, 60; scarlet fever, 33; diphtheria, 11.

At the present time there exists an epidemic of scarlet fever ever increasing and menacing the health and welfare of the community. Contagious diseases produce about 7 per cent. of the total mortality. The average mortality of scarlet fever is about 8½ per cent. In the Metropolitan Asylums' Board in London in 1890, with a total number of admissions of 10,343 the case mortality was only 2.97 per cent. The average mortality of measles in 12,273 cases admitted to the Glasgow Fever Hospital from 1870-89 was 8.8 per cent.

For diphtheria the London notifications for 1892 to 1897 show that more than one-third of the cases are under the age of 5 years, and rather less than a third are between the ages of 5 and 10. The mortality before the use of antitoxin was used 50 per cent. of the children under 5 years died. Since antitoxin is in use the London Fever Hospitals report for 1898 a mortality of 15.5 per cent. The complications arising from these diseases are varied and often so grave as to leave the child so disabled physically that he is unable to properly take up the struggle for existence.

We find that there exists a marvelous amount of ignorance among many of the community concerning the dangers arising from dissemination of these diseases. They are due to errors of thought which must be corrected:

1. It is considered by some that every child must sometime contract a contagious disease or infectious disease and, therefore, parents in many cases disregard all safeguards and in such diseases as varicella and measles invite infection.

2. The individual in his ideas of liberty, is of the opinion that it is his freedom to use his discriminating powers to neglect his own health and injure that of others; hence, in attempting to enforce quarantine, the health authorities are always embarrassed because where infectious diseases occur in families of the working class the economic argument of self protection from dependence and possible pauperdom through weeks' loss of time in isolation of quarantine outweighs the community good and therefore cases proceed to spread the disease at a greater cost to the community.

The municipality of Buffalo requires:

1. The notification of every case of contagious and infectious disease. In many instances people in fear of quarantine, if sending for a physician allow their children to go unattended, thus a focus of infection may be ever present for the dissemination of disease.

2. When such cases are thus reported, the Board of Health placards the house, especially for variola, scarlatina, diphtheria (measles should also be placarded); quarantine is maintained; the patient and all living in the same dwelling are isolated.

The records from the inspectors of the Health Department show that it is the rule rather than the exception for quarantine to be broken. So menacing has this proven that Health Commissioner Wende was obliged to order the Police Department to enforce quarantine.

Your commission is of the opinion that an army of police officers requiring three shifts of eight hours each would be necessary to properly and adequately enforce quarantine.

There are at present five medical school inspectors whose duty requires them to check the spread of contagious diseases in the schools. At this time it is an every-day occurrence that contagious diseases are discovered in the schools and excluded by the inspectors, but the number of medical school examiners to perform the work outlined for them is a mere fraction of what there should be. (Chicago has 110; New York city, 150; Rochester, 12; as compared to Buffalo's 5).

As at present conducted, quarantine rigidly enforced without proper facilities for the segregation and treatment of communicable diseases is a hardship on the following peoples:

1. If a case of infectious disease necessitating placarding occurs where shop and home are in the same building, business is stopped and the source of income interfered with.

2. If a case occur in a boarding or lodging house, the house is placarded and the business ruined.

3. If a domestic employee becomes infected, the house in which such employee lives is quarantined, the children are not allowed school attendance and the business of members of the family is interfered with.

4. If occurring in the family of a char woman, her means of livelihood is stopped.

5. If occurring in the family of wage-earners, their means of livelihood is stopped.

6. If as so often happens, the disease develops in a tenement, its spread under conditions of crowding and unsanitary environment is great because the enforcement of quarantine is difficult and impossible.

7. Travelers and residents in hotels in the city of Buffalo are at once placed in a dreadful plight; they must leave the hotel within an hour's notice or the hotel is placarded; the law forbids them to hire a public conveyance without first notifying the owner of the conveyance for the purpose for which it is to be used; the law forbids them to leave the city, or to go upon the public streets or in public places; they are not allowed to receive visitors. The hospitals are, as a rule, crowded and have no room for them.

The Erie County Hospital can accept 8 cases; the Sisters hospital, 10 cases. The General Hospital contagious pavilion was condemned as unsanitary by the health department without making provision elsewhere for the number of beds put out of commission. The Children's Hospital can accommodate 10 cases, but only young children. There are, then, in the city of Buffalo with nearly 400,000 inhabitants accommodations for 18 adult cases or 28 cases including the children. It was recently demonstrated that if the Erie County Hospital is filled with 8 cases of scarlet fever that, necessarily, there is no room for diphtheria and the same is true of other hospitals in the city, for the reason that they are not on the pavilion system.

The conditions therefore at present existing in the city of Buffalo are intolerable, unreasonable and impossible for the general welfare and public good, because there is not in Buffalo, as in other cities of its size and importance, a hospital conducted by the city for the segregation and scientific treatment of contagious diseases.

What some other cities have done. Glasgow was the first city to establish municipal infectious hospitals. In Glasgow 9.79 persons per 10,000 of inhabitants can be treated for infectious diseases. In London, Eng., the first municipal fever hospital was opened January 25, 1870. There are now 17 such hospitals situated in every part of London giving accommodations for 9 persons per 10,000 of inhabitants. Up to October, 1903, 3,264,272 pounds were expended for land and buildings. In 1871, 864 cases of scarlet fever were treated; in 1903, 18,316 scarlet fever cases were treated.

In 1871 the annual mortality per 1,000 inhabitants from scarlet fever was 0.58; in 1903, .08. In 1888, diphtheria had a mortality of .32 per 1,000; in 1903, .16 per 1,000 of total inhabitants. So satisfactory have the municipal hospitals proven that at present in the city of London 80 per cent. of all contagious diseases reported are treated in the fever hospitals.

In 1862 Boston, Mass., erected the first municipal hospital in this country. Boston now has a model hospital for contagious diseases and its resident physician occupies the chair on infectious diseases at Harvard College. Boston has 3.85 beds for each 10,000 inhabitants. Buffalo 1 bed for 13,330 and New York city has the following hospitals for the treatment of contagious and infectious diseases: Willard Parker Hospital, 125; Scarlet Fever Hospital, 392; Riverside Hospital, 335; Kingston Avenue Hospital, 366; Tuberculosis Sanatorium at Otisville, Orange Co., N. Y., 264; Reception Hospital, 23. New York city also maintains a hospital and dispensary for the treatment of contagious eye diseases and three clinics for the treatment of persons suffering from tuberculosis.

In Philadelphia about 30 per cent. of contagious diseases are treated in the Municipal Hospital. The present hospital facilities have been proven inadequate and a new municipal hospital is in the course of construction and almost completed. This hospital is on a plot of ground of seventy-five acres and has widely separated buildings. The total cost of this institution when completed will be in the neighborhood of $3,000,000.

Indianapolis is about to build a new contagious hospital at a cost of over $75,000. The plans and specifications are not yet completed. However, it is the intention to divide it into separate buildings connected with a single administration building.

The city of Milwaukee operates two hospitals for contagious disease. Cleveland, O., has at present under construction a new contagious disease building which is about finished. The city also has a tuberculosis sanatorium connected with the City Hospital and also a sanatorium at the Cooley Farm in Warrenville, O.

Cincinnati, O., is about to build a municipal hospital which will be one of the finest institutions in the country. The following are the buildings with their cost of the contagious group as given the committee by the architects Messrs. Samuel Hannaford & Sons: power and laundry building, $34,000; isolation and administration building, $100,000; Ward O, $55,000; Ward P, $56,000; Ward Q, $57,000; connecting tunnels and porches, $15,550; total, $337,500. This figure does not include heating and ventilating apparatus. The plans are here and from them you will readily see that Cincinnati has done Buffalo an excellent service in showing us how to build such a hospital worthy of the name.

Baltimore is now building an infectious hospital. The buildings are grouped about the administration building, having separate wards for the sexes and races. It will be located a short distance from the city on a separate tract of land of twenty acres. It is contemplated to eventually erect separate buildings for each of the more virulent minor contagious diseases. Chicago has just inaugurated a hospital for scarlet fever, diphtheria, whooping cough and measles. There are four buildings so that isolation is complete.

St. Louis has a special hospital for contagious diseases at the City Hospital, situated in one corner of the block on which the entire group of hospital buildings stand. It is disconnected from all the other buildings and separated from the nearest by 120 feet, having a capacity of 150 beds. The class of cases treated are measles, scarlatina, diphtheria and erysipelas.

Minneapolis, Minn., has a contagious building having separate wards for tuberculosis, erysipelas, measles, scarlet fever and diphtheria. Capacity, 60 patients; cost, $42,000. The hospital was a good one when it was built ten years ago but is out of date now.

St. Paul has a very fine contagious hospital. Pittsburg, Pa., has a municipal contagious hospital which in 1907 treated 602 cases and expended $28,532.45. There are other municipal hospitals in many other cities from which we have not received answers to our enquiries.

Shortly after the appointment of this commission, the Medical Society of the County of Erie appointed a committee of fifteen of which Dr. John D. Bonnar is chairman. Both committees have coöperated and worked as one. Together we framed suitable resolutions urging the establishment and maintenance of a municipal hospital for infectious diseases and presented them to the board of aldermen, Monday, October 5, 1908. These resolutions were referred to the Committee on Sanitary Affairs, of which Dr. James S. Porter is chairman, and a public hearing will be held in the Common Council Chamber, Thursday afternoon, at 2.30 P.M., October 29. The commission, desiring to ascertain the conviction of the medical profession upon the needs of a municipal hospital for contagious diseases, sent out return post cards and we desire to state that such a hospital is considered a necessity by the medical profession by almost a unanimity of expression.

As the commission desires that the public at large should give expression of its opinion upon so urgent a question and should be represented at the public hearing, we have addressed letters to various large and important bodies, such as the Chamber of Commerce, the Manufacturers' Club, the East Side Business Men's Association, the City Federation of Women's Clubs, and the like, and express pleasure at the hearty responses of coöperation. We invited the mayor, the board of aldermen, as well as representatives from these organisations to avail themselves of the privilege of hearing the address of our guest, Dr. Robert J. Wilson, who so magnanimously has placed himself at the disposal of your commission and the Buffalo Academy of Medicine.

The commission therefore comparing the present necessities of Buffalo, after a study of similar conditions in many other cities, urges the importance of the establishment of a municipal hospital for contagious diseases for 250 beds for the following reasons:

1. Such a hospital effectually removes the focus of infection and prevents the dissemination of other centers of infection.

2. The person affected is under medical supervision.

3. The precautions necessary to fully isolate a case from its inception to the termination of the disease require a vigilance not known to the layman or the general practitioner.

4. It allows the wage-earner to maintain his position.

5. It does not deprive the mother of her duties as housekeeper.

6. It permits the children not diseased in an affected family to attend school, provided the diseased is removed to the hospital and the place disinfected.

7. The mortality rates in hospitals is less than in the homes and the dangerous complications requiring instant treatment (tracheotomy, intubation) more readily attacked.

8. In hospitals not treating infectious or contagious diseases, it gives them a place for the immediate isolation of the patient. (The Municipal Hospital of Philadelphia cared in one year for 178 cases transferred from other hospitals in the general wards of which the disease developed.

9. Hotels, tenement houses, apartment houses, lodging and boarding houses, and homes where shop and home are combined, need not be placarded provided the patient is removed to the hospital and the apartment disinfected, thus business as formerly threatened with ruin and destruction may continue.

We submit this report in the hope that the Buffalo Academy of Medicine will use every means at its command for the establishment and maintenance of a municipal hospital for infectious diseases, and urge each member to be present at the public hearing, Thursday, October 29, at 2.30 P.M., at the council chamber.

Julius Ullman, Chairman
Allen A. Jones,
Grover W. Wende,
DeWitt H. Sherman,
Robert F. Sheehan, Jr.

